# The theory of tumor immuno-ecodynamics

**DOI:** 10.3389/fimmu.2026.1823415

**Published:** 2026-05-21

**Authors:** Xiaoping Chen

**Affiliations:** 1 State Key Laboratory of Respiratory Disease, Guangzhou Institutes of Biomedicine and Health, Chinese Academy of Sciences, Guangzhou, China; 2 CAS Lamvac (Guangzhou) Biomedical Technology Co., Ltd., Guangzhou, China

**Keywords:** immune equilibrium, immunodynamics, information theory, mathematical model, tumor ecodynamics, tumor ecosystem, tumor immuno-ecodynamics, tumor immuno-ecosystem

## Abstract

**Background:**

In previous studies, I established theoretical frameworks for immunodynamics and tumor ecodynamics, respectively, to quantitatively describe immune responses and the interactions between the immune system and the tumor ecosystem. Although these two theories are closely related, they are still distinct. This study aims to integrate these two theories into a unified theory termed tumor immuno-ecodynamics, and provide a simple standard mathematical model for medical researchers and clinicians.

**Methods and results:**

After conducting an information-theoretical analysis on the fundamental equations of immunodynamics and tumor ecodynamics, and incorporating them into the framework of information theory, I standardized a series of equations by subtracting the background values from the measured values of the variables. At the same time, I mathematized the cancer immunoediting theory that had been included in this framework, and rigorously proved some important formulae mathematically. Thus, a standard mathematical model termed the theory of tumor immuno-ecodynamics was established to unify the above two different theories. This model containing ten standard conceptual equations focuses on the signal (information) connections and interactions between the immune system and the tumor ecosystem, which are redefined as two subsystems of the tumor immuno-ecosystem.

**Conclusion:**

The standard mathematical model composed of these ten conceptual equations unifies immunodynamics and tumor ecodynamics into the theory of tumor immuno-ecodynamics, which incorporates four existing important theories, namely the theories of immune equilibrium, the tumor ecosystem, the cancer hallmarks, and the cancer immunoediting. This theory can quantitatively describe the actions of the tumor immuno-ecosystem, laying a theoretical foundation for the development of immuno-oncology from qualitative to quantitative science.

## 1 Introduction

In previous studies, I have initially established a series of mathematical equations for immunodynamics (1) and tumor ecodynamics (2). These equations can quantitatively describe immune responses, the interactions between the immune system and the tumor ecosystem, and have established new efficacy evaluation criteria for individualized cancer immunotherapy, which is termed the Response Evaluation Criteria in Solid Tumors for immunotherapy based on tumor ecodynamics (ieRECIST) (2). However, this earlier theory borrowed certain concepts from Newtonian mechanics, which may lead to certain misunderstandings in the biological field, especially in the fields of immunology and oncology. During the process of formulating the early theory, I utilized some basic concepts or definitions from Newtonian mechanics, such as action and reaction forces (braking force), velocity, and momentum. However, it is extremely difficult to measure the action force or reaction force, as well as the movement velocity and momentum of a biological molecule within a living organism. I merely borrowed these concepts to describe the intensity of the immune response, such as its intensity in acting on the tumor ecosystem, or conversely, the intensity of the effect of the tumor ecosystem on the immune system, and so on. Therefore, in this study, although some of the names used are still those from Newtonian mechanics, as well as some related terms derived from them, such as immunodynamic factors and immune force, etc., we must clearly express the biological connotations that these names refer to. Essentially, the immunodynamic factors mentioned previously (1) are biological information molecules, more precisely, they are information that regulate the immune responses. In this way, we can reanalyze the earlier theory from the perspective of information theory (3). For instance, the tumor ecological force is redefined as the information flow (intensity) of the inhibitory signals that the tumor ecosystem transmits to the immune system. The product of this information flow and the tumor volume is defined as the tumor ecological momentum. At the same time, we need to consistently use the adjusted values (2) obtained by subtracting the background values (the detection value of the normal tissue at the location where the tumor is situated before the tumor formation is termed the background value) from the detection values to establish a standard mathematical model. This avoids the deficiency in the earlier theory where the detection values were directly used to establish the mathematical model (2), which was mainly due to the convenience of clinical application and some other reasons. Therefore, the aim of this study is to establish a mathematically rigorous and precisely measured standardized mathematical model, in order to promote the theoretical development of immunodynamics and tumor ecodynamics. Moreover, although the cancer immunoediting theory (4, 5) has been incorporated into the framework of immunodynamics/tumor ecodynamics in the previous studies, it has not yet been mathematized, nor has an important formula for calculating the capacity of the immune system (immune capacity) been proposed, nor has the origin and mathematical proof of the cube-square ratio formula [appearing in the early paper (2)] been given. Therefore, in this study, all these issues will be addressed one by one. Through this study, I established 10 standard conceptual equations. These standard conceptual equations unified four important theories in immunology and oncology, namely the theories of immune equilibrium (6), the tumor ecosystem (7), the cancer hallmarks (8), and the cancer immunoediting. Furthermore, in this study, I proposed two additional new equations, namely the equations for calculating the comprehensive response rates of tumors, which replace the two less accurate equations used in the previous studies (2). However, these are not conceptual equations but belong to the category of application equations. Collectively, this study standardizes the previously established mathematical models that quantitatively describe the immune responses, the interactions between the immune system and the tumor ecosystem, and integrates them into a unified theory termed tumor immuno-ecodynamics.

This research lays the foundation for the transformation of immunology and oncology or immuno-oncology from qualitative to quantitative sciences. At the same time, it provides a clear-concept and mathematically simple theoretical model for medical researchers and clinicians, especially those in oncology practice, and promotes the application and popularization of this theory in clinical settings.

## 2 Methods and results

### 2.1 Information-theoretical analysis of immunodynamics and tumor ecodynamics

**Figure 1:**
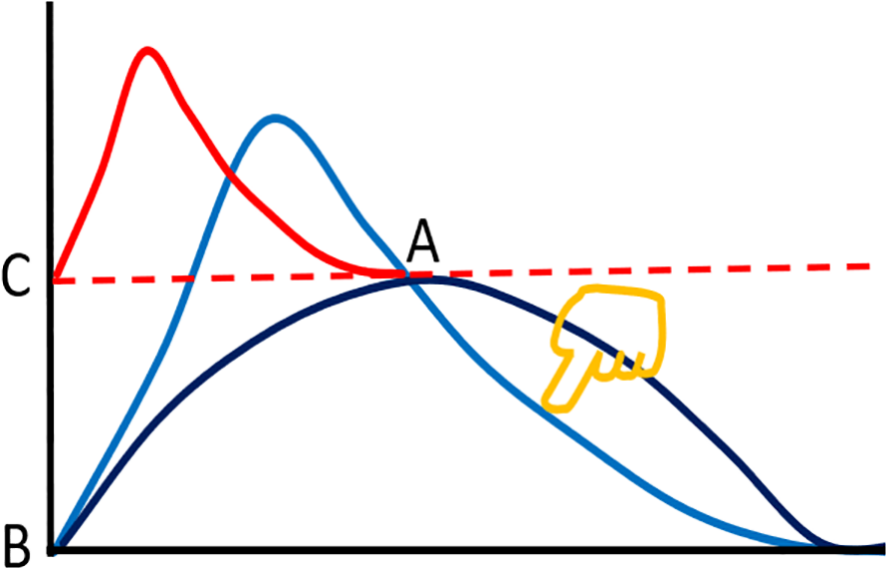
Mechanism diagram of immunodynamic process. It is assumed that the light blue curve representing the positive immune power ( $P_{pi}$) coincides with the dark blue curve representing the negative immune power ( $P_{ni}$) before the immune response. Initiating the immune response is equivalent to pressing the light blue curve with finger (golden yellow) from the upper right to the lower left to separate the two curves, forming three intersections, where two curves form from point A to point B, and the corresponding points of the two curves have different values in the ordinate. A series of ratios between them (the ratio of $P_{pi}$ to $P_{ni}$) produces an immune force ( $F_{im}$) curve from point C to point A (red), and the reciprocal of this series of ratios ( $P_{ni}$ to $P_{i}$) produces an immune braking force ( $F_{ib}$) curve from point C to point A (a curve that is the same in shape as the red curve but inverted vertically, omitted).

In the earlier research (1), I have already mathematized the theory of immune equilibrium (6), that is, pairing positive immunodynamic factors with negative immunodynamic factors one by one, such as X1/Y1, X2/Y2, X3/Y3… Xn/Yn, to obtain the following Equation 1, which is defined as the theoretical equation of immune force ( $F_{im}$). The numerator in the formula is defined as the positive immune power ( $P_{pi}$), and the denominator is defined as the negative immune power ( $P_{ni}$). At the same time, the reciprocal formula of this equation is defined as the theoretical equation of immune braking force ( $F_{ib}$), that is, the following Equation 2. Then it is assumed that before the immune response, the $P_{pi}$ curve (light blue curve in **Figure 1**) completely overlaps the $P_{ni}$ curve (dark blue curve in **Figure 1**), and the two immune powers are in balance. Initiating the immune response is equivalent to pressing the light blue curve with your finger, so that the two curves form three intersections (**Figure 1**), where the values of the two curves from point A to point B are different in the direction of the vertical axis, and plotting their ratios forms a red curve from point C to point A, which is the theoretical curve of $F_{im}$. This theoretical curve is consistent with the characteristics of immune response, that is, the rapid rise part of the curve, represents the stage of innate immune response; the slow decline part after the peak, represents the stage of adaptive immune response; and the final part (returning to the baseline level), represents the end of immune response. Next step, based on the functional data, from a series of hypothetical equations and their corresponding curves, a curve that is consistent with the theoretical curve is found, and the corresponding equation of this curve is defined as the practical equation of $F_{im}$, that is, the Equation 3 below, and its reciprocal formula is defined as the practical equation of $F_{ib}$, that is, the Equation 4 (1) below. Further step, using the method of reverse immunodynamics, the immune braking force is regarded as the tumor ecological force ( $F_{ib}$), and the immune force is regarded as the tumor ecological braking force ( $F_{im}$), and the product of $F_{ib}$ and tumor volume ( V) is defined as the tumor ecological momentum ( $M_{te}$), which is described by the Equation 5 (2) below.

(1) $$F_{im}=\frac{P_{pi}}{P_{ni}}\;=\frac{\left(X1)(X2)(X3)\ldots (Xn\right)}{\left(Y1)(Y2)(Y3)\ldots (Yn\right)}$$

(2) $$F_{ib}=\frac{P_{ni}}{P_{pi}}\;=\;\frac{\left(Y1)(Y2)(Y3)\ldots (Yn\right)}{\left(X1)(X2)(X3)\ldots (Xn\right)}$$

(3) $$F_{im}=\;\frac{\left(IFN\gamma )(pSTAT1\right)}{\left(TGF\beta )(pSTAT3\right)}$$

(4) $$F_{ib}=\;\frac{\left(TGF\beta )(pSTAT3\right)}{\left(IFN\gamma )(pSTAT1\right)}$$

(5) $$M_{te}=(F_{ib})(V)=\frac{\left(TGF\beta )(pSTAT3\right)}{\left(IFN\gamma )(pSTAT1\right)}(V)$$

As mentioned above, we must replace the measured values with adjusted values (indicated by a stroke on the upper right corner of the variable) (2) to establish the standard equations. Therefore, the above Equations 3–5 are rewritten as the following Equations 6–8. From here on, all necessary variables will use the adjusted values.

(6) $$F_{im}^{'}=\;\frac{\left(IFN\gamma^{\prime })(pSTAT1^{\prime }\right)}{\left(TGF\beta^{\prime })(pSTAT3^{\prime }\right)}\;\;\;$$

(7) $$F_{ib}^{'}=\frac{\left(TGF\beta^{\prime })(pSTAT3^{\prime }\right)}{\left(IFN\gamma^{\prime })(pSTAT1^{\prime }\right)}$$

(8) $$M_{te}^{'}=(F_{ib}^{'})(V)=\;\frac{\left(TGF\beta^{\prime })(pSTAT3^{\prime }\right)}{\left(IFN\gamma^{\prime })(pSTAT1^{\prime }\right)}(V)\;\;$$

As mentioned earlier, we can reanalyze the above theory from the perspective of information theory. According to the fundamental concept of information theory (9), the information entropy ( H, representing the degree of information disorder) of a group of unorganized information (immunodynamic factors, variable X) is the sum of the entropy of each information, which is defined as the following Equation 9, where p represents the probability of the information and log is the logarithm base 2. The greater the uncertainty of a variable, the greater the entropy, and the greater the amount of information required to make sense of it. If it is known that an information X is related to another information Y, for example, they are molecules on a signaling pathway, then the joint probability distribution (known as joint entropy) of their simultaneous occurrence can be calculated, as shown in Equation 10 below, and the probability distribution of X under the premise of different values of Y, namely the conditional probability distribution (known as conditional entropy) can also be calculated, as shown in Equation 11 below. It can be proved mathematically that H(X|Y)≤H(X), that is, with more information about Y, the uncertainty about X is reduced, and therefore its entropy H(X) is also reduced. Another important concept is relative entropy (or Kullback-Leibler divergence, denoted by  D), which measures the similarity of two functions with positive values, defined by the following Equation 12 (9). We can assume that a series of immunodynamic factors, when they are not organized, are like scattered sand, and their information entropy is additive, as shown in Equation 9, so the entropy is high. However, if they are paired, such as the immunodynamic factor pair ( X/Y), then their joint or conditional entropy is greatly reduced, as shown in Equations 10, 11. If you pair all the positive immunodynamic factors (positive information) with all the negative immunodynamic factors (negative information) one by one ( X1/Y1, X2/Y2, X3/Y3… Xn/Yn) (including the known pairs (1, 10–16) listed in **Table 1** and the unknown pairs), and hang X series factors on the light blue curve and Y series factors on the dark blue curve in order, then the immune force curve (red) formed by the interaction of these two series of factors has considered all possible immunodynamic factors, and they only represent a value (intensity), no longer have any other properties. That is, only their intensities (values) have effect on the immunodynamic curve. Mathematically, no matter how many factor pairs are added, they have little effect on changing the curves described by the Equations 6, 7 composed of the four core factors (two core factor pairs). In this way, all the immunodynamic factors originally with a very disordered distribution (large entropy or divergence) are transformed into a highly uniform information flow (entropy or divergence equals 0). If the theoretical Equation 1 is set as a function p, then its entropy H(p)=0. Similarly, if the practical Equation 6 is set as a function q, then H(q)=0. Therefore, the relative entropy of the two functions is also equal to 0, i.e., D(p||q)=0. Using the same method, it can be proved that the relative entropy between the theoretical Equation 2 and the practical Equation 7 is also equal to 0. Therefore, Equations 1, 6 are equivalent, and Equations 2, 7 are also equivalent. The above analysis results are highly consistent with those of my previous research (1). For instance, the theoretical equation of $F_{im}$, namely Equation 1 mentioned above, already contains an infinite number of positive and negative factor pairs. The curve it describes is consistent with that described by the practical equation with only two core factor pairs, namely Equation 3, which proves that Equation 3 can represent Equation 1.

**Table 1:** Positive and negative immunodynamic factor pairs *.

Positive	Negative	Sort module
pSTAT1	pSTAT3	JAK-STAT phosphorylation axis
pSTAT4	pSTAT6	
JAK	SOCS	
IFNα/β	SOCS1/3	
IFNγ	TGFβ	Regulation of cytokine polarity
IL-2	TGFβ	
IFNγ	IL-10	
IL-12	IL-27	
RIG-I	LGP2	Intracellular nucleic acid perception
STING	PTP1B	
NKp30	KIR2DL1	NK/myeloid activation-inhibitory receptors
NKG2D	NKG2A	
Ly49D	Ly49A	
PIR-A	PIR-B	
DNAM-1	TIGIT	T-cell co-stimulation & immune checkpoints
CD28	CTLA-4	
ICOS	PD-1	
OX40	TRAIL-R	
CD40	Fas	
c-Myc	Foxp3	Transcriptional axis of T cell fate determination
NF-kB	IκBα	TLR/inflammatory core transcription pathway
MyD88	TOLLIP	
TLR	SIGIRR	
BCR	CD22	B cell receptor homeostasis
mTORC1	AMPK	Immune metabolism & transcriptional fate axis
M1	M2	Myeloid cell functional phenotype
…	…	…

*Pairing based on signal pathways and/or action mechanisms (sort module).

**Figure 2:**
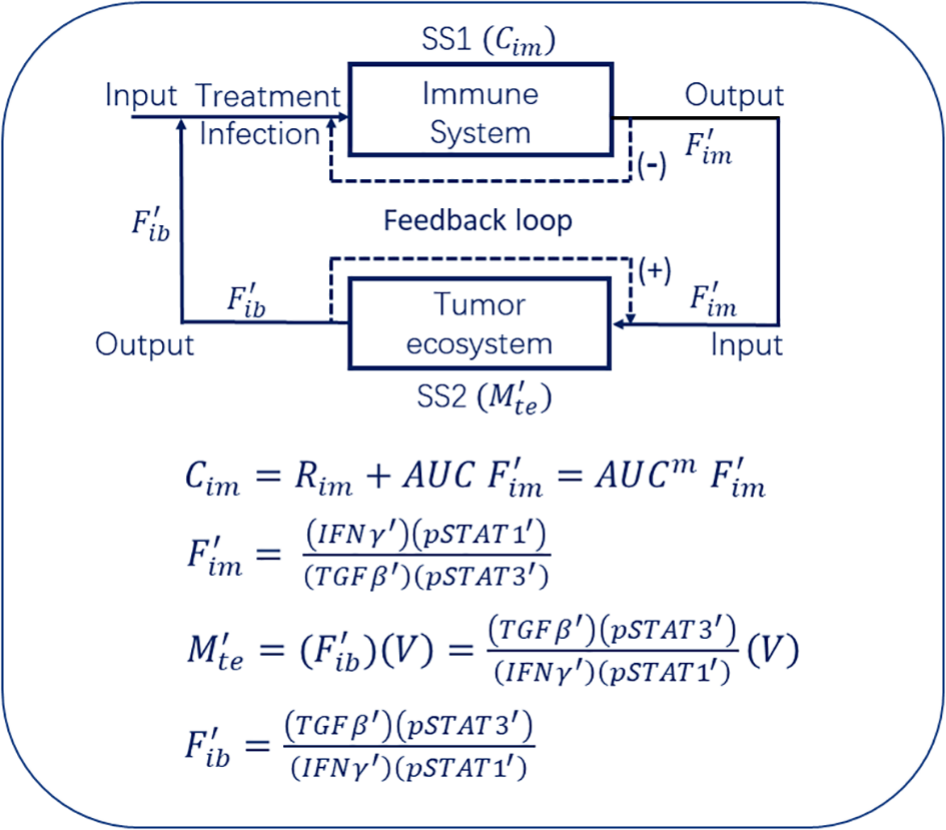
The model of tumor immuno-ecosystem. The model links the immune system (SS1) quantified at $C_{im}$ to the tumor ecosystem (SS2) quantified at $M_{te}^{'}$ through $F_{im}$ and $F_{ib}$ transmissions.

The core content of information theory is information compression, transmission and storage (3, 17). According to the above analysis, we can set that the positive information hanging on the $P_{pi}$ curve (light blue, **Figure 1**), and the negative information hanging on the $P_{ni}$ curve (dark blue, **Figure 1**), are equivalent to compressing them into the two curves respectively; and then, using an algorithm, such as Equation 6, we can further compress the two types of information into one curve (red, **Figure 1**). This process is called information (or data) compression. The compressed information $F_{im}^{'}$ can then be transmitted from subsystem 1 (SS1, the immune system, **Figure 2**) to the subsystem 2 (SS2, the tumor ecosystem, **Figure 2**). Similarly, the compressed information $F_{ib}^{'}$ can also be transmitted from subsystem 2 (SS2, the tumor ecosystem, **Figure 2**) to the subsystem 1 (SS1, the immune system, **Figure 2**). This process is known as information transmission. Finally, the accumulation of $F_{ib}^{'}$ into the area under the $F_{ib}^{'}$ curve ( $AUC\;F_{ib}^{'}$) is equivalent to storing the information in a plane (see later section). Then, through another algorithm, such as Equation 8, the information stored in the plane ( $AUC\;F_{ib}^{'}$) is further converted into the information stored in space ( $M_{te}^{'}$, see later section). Therefore, the immunodynamics and tumor ecodynamics can be incorporated into the theoretical framework of information theory (**Figure 2**).

(9) $$H(X)=-\sum_{x\in X}P(x)logP(x)$$

(10) $$H(X,\;Y)=-\sum_{x\in X,\;y\in Y}P(x,\;y)logP(x,y)$$

(11) $$H(X|Y)=-\sum_{x\in X,\;y\in Y}P(x,\;y)logP(x|y)$$

(12) D(p||q)=∑x∈X P(x)·logp(x)q(x) 

### 2.2 The standard mathematical model termed the theory of tumor immuno-ecodynamics

The essence of immunity is homeostasis (18, 19), which can also be called the equilibrium (6). Immunodynamics is the mathematical description of this essential property. Whether it is an infection or a tumor, through the immune response to clear the pathogens and tumor cells, the immune system returns to the homeostasis (equilibrium) before the onset of the disease. At the same time, in the study of tumor ecodynamics, I have explained that tumor ecosystem is a structural concept, and cancer hallmarks (including 14 known cancer hallmarks) are the functional phenotypes of this system (2). These functional manifestations are associated with $F_{ib}^{'}=\frac{\left(TGF\beta^{\prime })(pSTAT3^{\prime }\right)}{\left(IFN\gamma^{\prime })(pSTAT1^{\prime }\right)}$ signaling, so the tumor ecological force curve reflects the information flows of the tumor ecosystem.

First, I included the whole tumor ecosystem into the category of negative immunity, thus forming an antagonistic unity between positive immune power $P_{pi}^{'}=(IFN\gamma^{\prime })(pSTAT1^{\prime })$ and negative immune power $P_{ni}^{'}=(TGF\beta^{\prime })(pSTAT3^{\prime })$, which is the prototype of the system to be established in this study. The outputs of this prototype are $P_{pi}^{'}$ and $P_{ni}^{'}$. When the outputs of the system are adjusted, that is, the positive immune power $P_{pi}^{'}$ is adjusted to the immune force $F_{im}^{'}$, and the negative immune power $P_{ni}^{'}$ is adjusted to the tumor ecological force $F_{ib}^{'}$, the tumor ecosystem is then formally independent from the immune system, so that the tumor ecosystem and the immune system constitute an opposing unity of mutual restriction. As mentioned earlier, $F_{im}^{'}$ and $F_{ib}^{'}$ represent the information flow between the immune system and the tumor ecosystem. Therefore, we can incorporate immunodynamics and tumor ecodynamics into the framework of information theory, thereby establishing a standard mathematical model termed the theory of tumor immuno-ecodynamics to describe the tumor immuno-ecosystem that contains the two subsystems, namely the immune system (SS1, quantified at $C_{im}$, will be discussed later) and the tumor ecosystem (SS2, quantified at $M_{te}^{'}$, will be discussed later) (**Figure 2**). Obviously, this model indicates that the immune system controls (suppresses) the tumor ecosystem through $F_{im}^{'}$ signal, while the tumor ecosystem controls (suppresses) the immune system through $F_{ib}^{'}$ signal. Meanwhile, this model has linked infection immunity with tumor immunity (**Figure 2**). The following conclusions or inferences can be drawn from this model. (1) Whether an infection (designated acute or subacute infection) can be used to treat cancer, or whether it can be used as an immunotherapy of cancer, depends on whether the infection stimulates SS1 to produce $F_{im}^{'}$, and the magnitude of its effect depends on the area under the curve of $F_{im}^{'}$ ( $AUC\;F_{im}^{'}$), namely the storage or accumulation of $F_{im}^{'}$. Researches by my team have demonstrated that *Plasmodium* infection can be used as an immunotherapy (*Plasmodium* immunotherapy) for cancer (20–31) (NCT02786589, NCT3375983, NCT03474822, NCT03375983, NCT05924776), because it fully activates the immune system through stimulating $IFN\gamma^{\prime }$ and $pSTAT1^{\prime }$ signals, while targeting/inhibiting the entire tumor ecosystem through blocking $TGF\beta^{\prime }$ and $pSTAT3^{\prime }\;$signals in tumor tissues (1, 2, 31, 32). More importantly, it induces strong $F_{im}^{'}$ and maintains $F_{im}^{'}$ for a long time (1), so the $AUC\;F_{im}^{'}$ value induced by this parasite is large enough. Based on this model, we can also understand the mechanisms of *Plasmodium* immunotherapy in this way: It activates the immune system to transmit $F_{im}^{'}$ to the tumor ecosystem, while inhibiting the tumor ecosystem to prevent it from transmitting $F_{ib}^{'}$ to the immune system. The immunotherapy that exerts its antitumor effect through this mechanism can be defined as immune ecotherapy or immuno-ecotherapy. Other parasitic infections should have similar anticancer mechanisms (33, 34). The bacterial infection and bacterial toxin used by William Coley, who is recognized as the father of cancer immunotherapy (35, 36), should also induce $F_{im}^{'}$ and $AUC\;F_{im}^{'}$. BCG vaccine therapy for bladder cancer (37) should have the same or similar actions. Some viral infections (such as oncolytic viruses) (38, 39) or virus-based therapeutic cancer vaccines (40) can also be regarded as immunotherapy, but virus is usually quickly eliminated by antiviral immunity (41), the maintenance time of induced $F_{im}^{'}$ should be short, and thus the $AUC\;F_{im}^{'}$ value should be small, so the effectiveness of the treatment alone may be limited. (2) Currently widely used immune checkpoint inhibitors (42, 43) can reactivate tumor-specific T cells, especially T cells in tumor tissues, thereby killing a part of tumor cells, and the dead tumor cells release not only tumor antigens and tumor-associated antigens, but also damage-associated molecular patterns (DAMPs), which further activate the innate and adaptive immunity (44), thereby inducing $F_{im}^{'}$ and $AUC\;F_{im}^{'}$. Principally, the therapeutic effects of immune checkpoint inhibitors depend on the sizes of $F_{im}^{'}$ and $AUC\;F_{im}^{'}$ induced by them. (3) The system has two feedback loops. SS1 can output $F_{im}^{'}$ during treatment or infection, to overcome negative immune power $\left(TGF\beta^{\prime })(pSTAT3^{\prime }\right)$ to promote immune response through positive immune power $\;(IFN\gamma^{\prime })(pSTAT1^{\prime })$, but due to the negative feedback mechanism of immune balance, SS1 eventually returns to the state of immune equilibrium. That is, $F_{im}^{'}$ returns to the baseline level before the immune response, which is a typical negative feedback loop [the dashed arrow of SS1, denoted by the symbol (-), **Figure 2**]. Therefore, any immunotherapy should be discontinued at or before $F_{im}^{'}$ returns to baseline levels. Otherwise, it will only increase side effects without increasing efficacy. SS2 (the tumor ecosystem) will form a positive feedback mechanism during the development of immune evasion, resulting in a vicious circle, because $TGF\beta^{\prime }$ and $pSTAT3^{\prime }$ are signaling molecules upstream and downstream of each other (45, 46), which is a typical positive feedback loop [dashed arrow of SS2, represented by the symbol (+), **Figure 2**]. Once this feedback loop appears, it cannot be checked by its own balancing mechanism and must be stopped by external therapeutic intervention.

**Figure 3:**
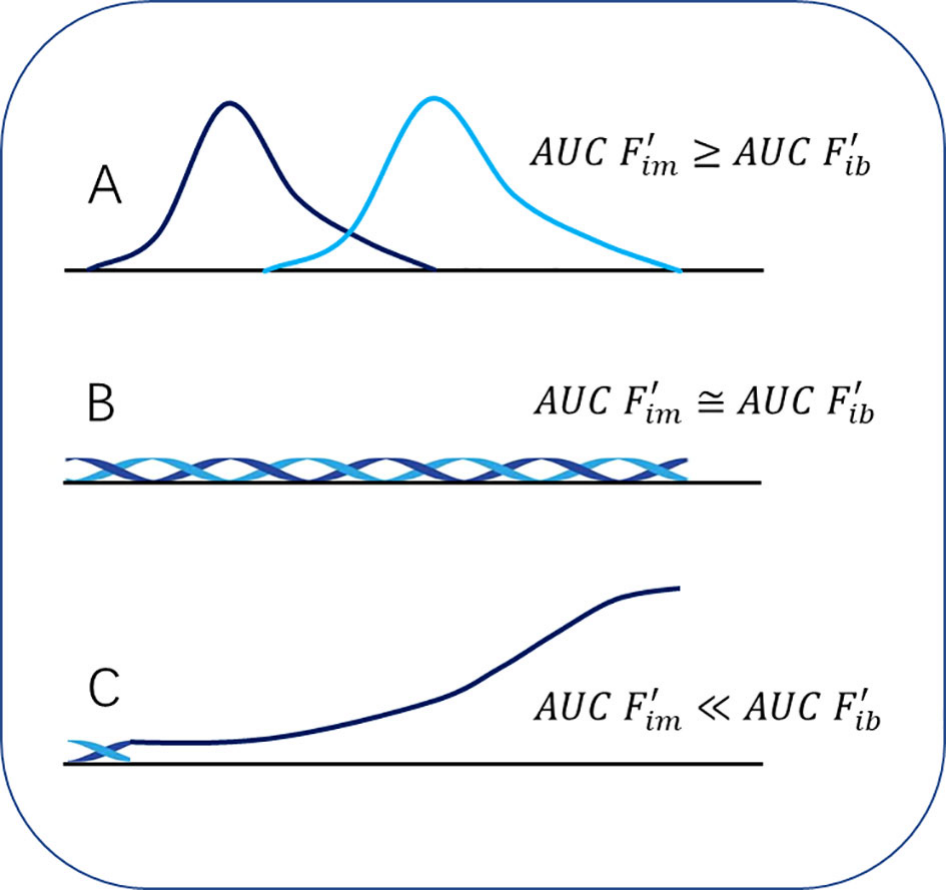
Mathematization of immunoediting theory. **(A)**, the stage of Elimination; **(B)**, the stage of Equilibrium; **(C)**, the stage of Escape.

With this model, the theory of cancer immunoediting (4, 5, 47) can be mathematized, and the mathematized cancer immunoediting theory can be regarded as a part of the model. According to the model, $F_{im}^{'}$ is stored or accumulated in $AUC\;F_{im}^{'}$; Similarly, $F_{ib}^{'}$ is stored in $AUC\;F_{ib}^{'}$. Therefore, the three stages of the cancer immunoediting can be described by mathematical expressions of the relationship between $AUC\;F_{im}^{'}$ and $AUC\;F_{ib}^{'}$. (1) In the stage of Elimination, the appearance of cancer cells is manifested as a dynamic $F_{ib}^{'}$ process, and $F_{ib}^{'}$ acts on the normal immune system (SS1) to initiate the immune response, which is manifested as a dynamic $F_{im}^{'}$ process (light blue curve in **Figure 3A**) following the dynamic $F_{ib}^{'}$ process (dark blue curve in **Figure 3A**). The negative feedback mechanism activated by $F_{im}^{'}$ causes $F_{im}^{'}$ to return to the baseline level at the end of the response. This process can be described by the following Equation 13. In this process, if $AUC\;F_{im}^{'}\geq AUC\;F_{ib}^{'}$, the cancer cells are completely eliminated, then the reaction is over, and the “patient” fully recovers. However, if $AUC\;F_{im}^{'}<AUC\;F_{ib}^{'}$, it will enter the Equilibrium stage. (2) In the Equilibrium stage, the immune system and the tumor ecosystem are evenly matched, which can be shown in **Figure 3B** (the dark blue curve represents the dynamic change of $\;F_{ib}^{'}$, and the light blue curve represents the dynamic change of $F_{im}^{'}$), and its mathematical expression is Equation 14 as below. At this stage, the dynamic changes of the $F_{im}^{'}$ and $\;F_{ib}^{'}$ curves may actually be irregular, but regardless of whether they are regular or irregular, the final result is $AUC\;F_{im}^{'}≅AUC\;F_{ib}^{'}$, that is, they are in dynamic equilibrium, not in a resting state. The two curves with equal AUC in **Figure 3B** describe exactly this dynamic equilibrium process. (3) When the tumor ecosystem overcomes $\;(IFN\gamma^{\prime })(pSTAT1^{\prime })\;$through $\left(TGF\beta^{\prime })(pSTAT3^{\prime }\right)$ and enters a positive feedback loop and vicious cycle, the Escape phase is announced. This process can be shown in **Figure 3C** (dark blue curve represents the dynamic change of $F_{ib}^{'}$), which is expressed by the following Equation 15.

(13) $$AUC\;F_{im}^{'}\geq AUC\;F_{ib\;\;\;}^{'}$$

(14) $$AUC\;F_{im}^{'}≅AUC\;F_{ib\;\;\;\;\;\;\;\;\;}^{'}$$

(15) $$AUC\;F_{im}^{'}\ll AUC\;F_{ib\;\;}^{'}$$

However, regardless of the stage (Elimination, Equilibrium or Escape) of the tumor ecosystem, its magnitude can be expressed by $AUC\;F_{ib}^{'}$. It is very important that $AUC\;F_{ib}^{'}$ can be transformed and stored as $M_{te}^{'}$. Therefore, it can be considered that $AUC\;F_{ib}^{'}$ and $M_{te}^{'}$ are equivalent, that is, $AUC\;F_{ib}^{'}=M_{te}^{'}$. This equation describes how the information flow $F_{ib}^{'}$ of the tumor ecosystem, after overcoming the flow $F_{im}^{'}$ of the immune system, continuously promotes the development of the tumor ecosystem, thereby forming a tumor ecosystem with a quantitative value of $M_{te}^{'}=(F_{ib}^{'})(V)$ at a certain point in time. This tumor ecosystem initially releases low levels of $F_{ib}^{'}$ to the immune system when the tumor begins to form. As the tumor ecosystem develops, the quantity of $F_{ib}^{'}$ gradually increases. Therefore, $F_{ib}^{'}$ forms a gradually ascending dynamic curve on the time axis. When it reaches the aforementioned time point, the cumulative value of $F_{ib}^{'}$ is the area under this curve ( $AUC\;F_{ib}^{'}$), and this area represents the tumor ecological momentum $M_{te}^{'}$ at that time point. As mentioned earlier, the tumor ecological momentum at this point time is $M_{te}^{'}=(F_{ib}^{'})(V)$, therefore $M_{te}^{'}=(F_{ib}^{'})(V)=AUC\;F_{ib\;}^{'}$. We can also conduct further derivations on this equation, namely, the tumor ecosystem with a magnitude of $M_{te}^{'}$ driven by a certain magnitude of $AUC\;F_{ib}^{'}$ requires an equivalent magnitude of $AUC\;F_{im}^{'}$ to completely dissolve it. This description is expressed in the following Equation 16. This step is particularly important because the start time of $F_{ib}^{'}$ is not known clinically and therefore it is not possible to calculate $AUC\;F_{ib}^{'}$. Using $M_{te}^{'}$ for $AUC\;F_{ib}^{'}$ is equivalent to using space to replace time, since $M_{te}^{'}$ contains the tumor volume V (which can be understood as space), i.e., $M_{te}^{'}=(F_{ib}^{'})(V)$. In other words, the tumor ecological momentum $M_{te}^{'}$ in all tumor tissues and blood system represents the entire tumor ecosystem, therefore, the larger the $M_{te}^{'}$, the greater the $F_{ib}^{'}$ transported to the immune system (**Figure 2**), and the greater the degree of immune suppression or immune tolerance. Thus, the competition between the tumor ecosystem (SS2) and the immune system (SS1) becomes the $M_{te}^{'}/AUC\;F_{im}^{'}$ ratio, which is defined as the moving cube-to-force square ratio, or cube-square ratio, expressed by $R_{cs}$, i.e., $R_{cs}=M_{te}^{'}/AUC\;F_{im}^{'}$, or $AUC\;F_{im}^{'}=M_{te}^{'}/R_{cs}$ (2). $M_{te}^{'}$ can be further indicated for pre-treatment ( $M_{te}^{'\Delta }$) or post-treatment ( $M_{te}^{'\nabla }$), so that we can calculate the individual disease remission rate $R_{te}$ obtained by immunotherapy, see the Equation 17 below. In addition to calculating remission rates and establishing new efficacy criteria (ieRECIST) for cancer immunotherapy, the expansion of Equation 17 can also be used to distinguish pseudoprogression, progression, and hyperprogression of tumors (2). If you know the $M_{te}^{'\Delta }$ before treatment, you can also calculate how much of the $AUC\;F_{im}^{'}$ is needed to achieve a desired therapeutic effect (e.g., $R_{te}\;=\;100\%$). Thus, the expression of $AUC\;F_{im}^{'}$ is the following Equation 18.

(16) $$AUC\;F_{im}^{'}=M_{te}^{'}=AUC\;F_{ib}^{'}$$

(17) $$R_{te}=(M_{te}^{'\Delta }\;-\;M_{te}^{'\nabla })/M_{te}^{'\Delta }$$

(18) $$AUC\;F_{im}^{'}\;(R_{te})=M_{te}^{'\Delta }/R_{cs}$$

The above Equations 17, 18 indicate that when $R_{te}=100\%$ (cure, regardless of clinical cure or biological cure, meaning complete removal of the tumor ecosystem), then the smaller the tumor ecological momentum, the smaller the $AUC\;F_{im}^{'}$ value required. This suggests that before immunotherapy, if you first reduce the burden of large tumors (through surgery or precision radiotherapy, etc.), immunotherapy can receive better results. At the same time, these two equations also show that the cure does not require the disappearance of the tumor, but as long as the tumor activity disappears (when $F_{ib}^{'}=0$, then $\;M_{te}^{'}=0$), it can be defined as a cure: for example, the tumor activity disappears ( $F_{ib}^{'}=0$), leaving scar tissue at the original tumor site. Such “living with the tumor” is essentially a form of cure. This equation also explains the results of our previous longitudinal analysis of global epidemiological data, which showed a significant inverse association between global malaria incidence and overall global cancer mortality (48). Because *Plasmodium* infection induces SS1 to produce $F_{im}^{'}\;$ (1), and only a small amount of $AUC\;F_{im}^{'}$ is needed to cure tumors in the immunoediting Equilibrium stage, or even very early clinical tumors (because $M_{te}^{'\Delta }$ is small). It can be hypothesized that patients with subclinical and very early clinical tumors are present in malaria-endemic areas, and their tumors should disappear after infection with *Plasmodium* parasites. Particularly, in malaria-endemic areas of Africa, *Plasmodium falciparum* expresses a protein termed VAR2CSA that could combine with various tumors (49). This may cause infected red blood cells to target tumor tissues, triggering immune responses in the tumors. Therefore, a negative relationship between global malaria incidence and cancer mortality has been shown in the population level (48).

As mentioned above, the tumor ecosystem (SS2) can be quantified as $\;M_{te}^{'}$ (Equation 8, **Figure 2**). But we have not yet defined the total capacity of the immune system (SS1). In my previous published paper, I have quantified “immunity”, that is, immunity = immune reserve + immune power (1). With this concept, we can further define the total capacity of the immune system (SS1) as the following Equation 19 (**Figure 2**).

(19) $$C_{im}=R_{im}+AUC\;F_{im}^{'}=AUC^{m}\;F_{im}^{'}$$

Where $C_{im}$ is the total capacity of the immune system (immune capacity), $R_{im}$ is the immune reserve, and $AUC^{m}\;F_{im}^{'}$ represents the maximum $AUC\;F_{im}^{'}$ that can be produced by the immune response. This equation suggests that: (1) If a cancer patient has received many courses of treatment or multiple treatments that impair immune function, their immune reserve and immune capacity must be significantly reduced, and the effect that any immunotherapy can achieve is quite limited (limited $AUC^{m}\;F_{im}^{'}$). (2) Although immune reserve or immune capacity is sufficient, if there is no immune response ( $AUC\;F_{im}^{'}=0$, or $C_{im}=R_{im}$, namely, there is no output of $F_{im}^{'}$ from SS1 to SS2), or no immunotherapy, then the immune system may not play an anticancer role. (3) If you want to maximize the antitumor effect, you need to fully activate the immune system (close to $AUC^{m}\;F_{im}^{'}$), of course, you also need to consider the balance between efficacy and side effects.

**Figure 4:**
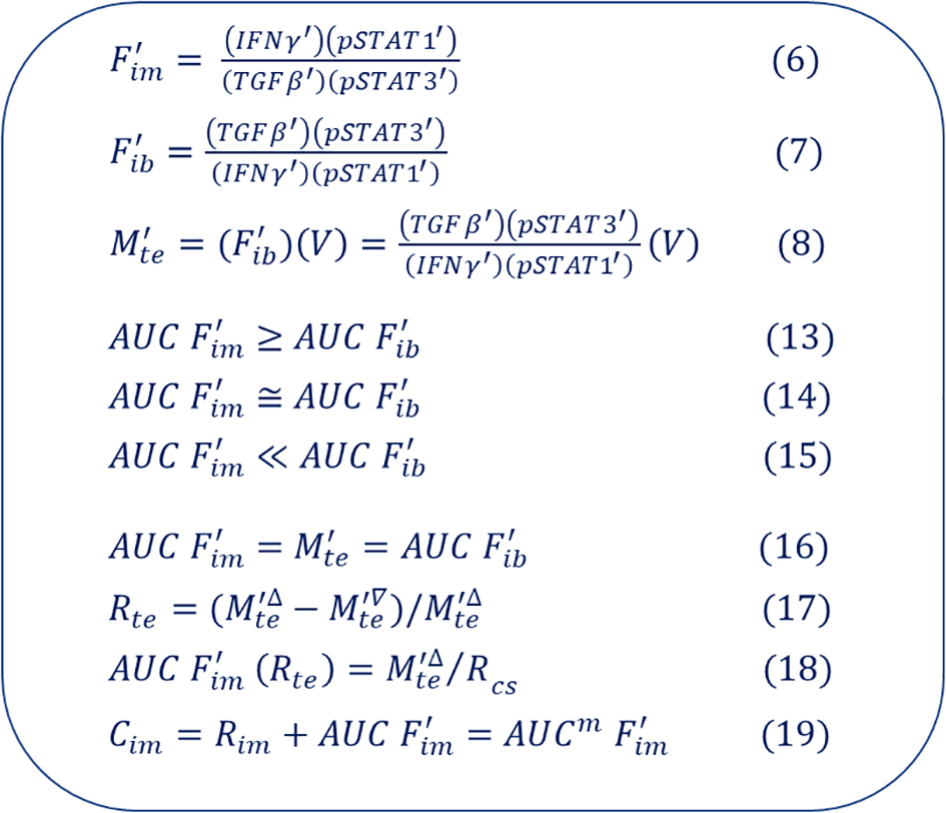
Standard mathematical model of tumor immuno-ecodynamics. This model is composed of ten standard conceptual equations.

The model formed by the above 10 equations, namely Equations 6–12, 13–19 should be the most minimalist mathematical model to describe the immune system, the tumor ecosystem and their interactions to date (summarized in **Figure 4**). Obviously, this model redefines the immune system and the tumor ecosystem as its two subsystems, and quantitatively describes the integrated tumor immuno-ecosystem (**Figures 2**, **3**). Therefore, this model represents the theoretical framework of the tumor immuno-ecodynamics that this study intends to establish.

It is necessary to emphasize that all abovementioned 10 Equations are only standard conceptual equations. However, merely having conceptual equations is not sufficient. We also need to establish standard equations that are convenient for clinical use. Standard application equations can be established based on the standard conceptual equations and the application equations established in previous study (2). These application equations mainly include indicators of tumor immuno-ecodynamics for the main tumor lesion foci and the patients’ blood samples. The transformation of these equations can be simply achieved by adding the symbol ( m) for the main lesion and the symbol ( b) for the blood sample after the names of each standardized variable. Therefore, this transformation does not need to be elaborated in this article. Only the following examples are needed. For instance, the total $AUC\;F_{im}^{'}$ cannot be calculated clinically, only the $AUC\;F_{im}^{'}$ of the blood specimens (denoted as $AUC\;F_{imb}^{'}$) can usually be calculated, and the $AUC\;F_{imb}^{'}$ represents only a fraction of the total $AUC\;F_{im}^{'}$, namely, a percentage (represented here by the weight $W_{b}$) of the total $AUC\;F_{im}^{'}$. In the same way, the $AUC\;F_{im}^{'}$ of the main tumor lesion (denoted as $AUC\;F_{imm}^{'}$) is also a percentage (expressed by the weight $W_{m}$) of the total $AUC\;F_{im}^{'}$. In this way, according to Equation 16, we can derive the more accurate comprehensive individual disease remission rate ( $R_{com}$), namely the Equation 20 below, and the comprehensive area under the immune force curve [ $AUC\;F_{imb}^{'}(R_{com}$)] required to achieve a certain comprehensive remission rate, namely the Equation 21, which are compatible with Equations 17, 18, and can be used in clinical practice. With these two more precise equations, we no longer need to combine PET-based parameters with immunodynamic factor-based parameters, but should apply them separately. That is, the Equations 17, 22 in my previous published article (2) should be replaced by the Equations 20, 21 in this paper, where $R_{tem}$ represents the remission rate of the main lesion, and $R_{teb}$ represents the remission rate of the blood, respectively. Of course, the experience values or value ranges of $W_{m}$ and $W_{b}$ in these equations need to obtain through clinical research and practice.

(20) $$R_{com}=\;(R_{tem})(W_{m})+(R_{teb})(W_{b})$$

(21) $$AUC\;F_{im}^{'}\;(R_{com})=[AUC\;F_{imm}^{'}\;(R_{tem})][W_{m}]+\;[AUC\;F_{imb}^{'}\;(R_{teb})][W_{b}]$$

It should be noted that $W_{m}$ in Equations 20, 21 is the same variable, and so is $W_{b}$. The proof of this conclusion is as follows: Let $W_{m}$ in Equation 20 be $W_{m1}$, and $W_{m}$ in Equation 21 be $W_{m2}$. According to Equation 16, $AUC\;F_{im}^{'}=M_{te}^{'}$, and through multiplying the variables on both sides of the equation by their respective weights, we can get $\left(AUC\;F_{im}^{'})(W_{m1})=(M_{te}^{'})(W_{m2}\right)$. Since $AUC\;F_{im}^{'}=M_{te}^{'}$, then $W_{m1}=W_{m2}\;$; that is, the $W_{m}$ of Equations 20, 21 is the same variable. Similarly, $W_{b}$ in both equations is the same variable. In addition, $W_{m}+W_{b}=1$.

## 3 Discussion

The standard mathematical model of tumor immuno-ecodynamics is derived from the unification of immunodynamics (1) and tumor ecodynamics (2), which are originated from the integration of four important theories in immunology and oncology (or immuno-oncology), namely the theories of immune equilibrium (6), tumor ecosystem (7), cancer hallmarks (8, 50) and cancer immunoediting (5). The thought method and logical process is to integrate the tumor ecosystem into the immune system, and then formally separating it from the immune system. The first step is to mathematize the theory of immune equilibrium and establish the theory of immunodynamics. In the second step, using the method of reverse immunodynamics, the immune force is regarded as the tumor ecological braking force, and the immune braking force is regarded as the tumor ecological force, which is equivalent to loading the entire tumor ecosystem into the negative part of the immune system. Because $P_{ni}^{'}=(TGF\beta^{\prime })(pSTAT3^{\prime })$, the core element representing negative immunity (or negative immune regulation), involves many seeming “non-immune” factors and is directly related to the most parts of the fourteen functional phenotypes (cancer hallmarks) of the tumor ecosystem (2), it is possible to incorporate all factors that are seemingly unrelated to immunity into the negative part of the immune system. The output of negative immunity (or negative immune regulation) is $P_{ni}^{'}=(TGF\beta^{\prime })(pSTAT3^{\prime })$, while the output of positive immunity (or positive immune regulation) is $P_{pi}^{'}=(IFN\gamma^{\prime })(pSTAT1^{\prime })$. In the third step, after adjusting the outputs of the system from $P_{ni}^{'}$ and $P_{pi}^{'}$ to $F_{ib}^{'}$ and $F_{im}^{'}$, the tumor ecosystem is formally independent from the immune system, the system is then separated into two subsystems, namely the antithetical unity of the tumor ecosystem (SS2) and the immune system (SS1). Therefore, the two subsystems form a contradictory entity, namely the tumor immuno-ecosystem (**Figure 2**) through the link of $F_{ib}^{'}$ and $F_{im}^{'}$. There are two important reasons to adjust the outputs. (1) $P_{ni}^{'}=(TGF\beta^{\prime })(pSTAT3^{\prime })$ and $P_{pi}^{'}=(IFN\gamma^{\prime })(pSTAT1^{\prime })$ are only the total outputs of the subsystems, while their valid outputs are $F_{ib}^{'}=P_{ni}^{'}/P_{pi}^{'}$ and $F_{im}^{'}=P_{pi}^{'}/P_{ni}^{'}$. (2) If the above adjustments are not made, the immune system of cancer patients must be logically redefined, that is, only the positive part of the system is remained, while the negative part has been subsumed into the tumor ecosystem, which will cause logical contradictions. Only by adjusting the outputs to $F_{im}^{'}$ and $F_{ib}^{'}$ can we restore the original pattern of the immune system, which consists of two opposing parts, namely the positive and negative parts ( $P_{pi}^{'}/P_{ni}^{'}$). At the same time, the tumor ecosystem also contains the positive and negative parts ( $P_{ni}^{'}/P_{pi}^{'}$). If you really think about this, you would find that it is actually an elaborate thought experiment. Now, we can interpret the tumor ecosystem in this way: because it is a very complex system, not easy to understand, it needs to be summarized as the cancer hallmarks (functional phenotypes) in order to elucidate its mechanism of action (51, 52). If the cancer hallmarks are further simplified to the subsystem of outputting $F_{ib}^{'}$, its mechanism can be further simplified to a mathematical equation, that is, $F_{ib}^{'}=P_{ni}^{'}/P_{pi}^{'}$. Finally, the above system is incorporated into the framework of information theory, and uniform adjusted values are used, to establish the standard mathematical model of tumor immuno-ecodynamics. Therefore, the most basic mathematical notations of this model are the four immunodynamic factors or information molecules ( $IFN\gamma^{\prime },\;\;pSTAT1^{'},\;\;TGF\beta^{\prime },\;\;pSTAT3^{\prime }$) and one oncology indicator ( V). The three equations constructed by these five symbols, namely Equations 6–8, are very easy to understand for immunologists and oncologists (or immuno-oncologists) as well as oncology clinicians. Equations 13–15 derived from Equations 6, 7 are simple model of the mathematization of cancer immunoediting theory. Equation 16 is an equivalent equation derived from Equations 6–8. Equations 17, 18, which are derived from Equation 8 and the equivalent Equation 16, can be used to establish a new therapeutic efficacy evaluation system (ieRECIST) for personalized cancer immunotherapy when used in conjunction with the application equations (2). It should be point out that, in this study, I propose a new equation, namely Equation 19, for quantitative description of the immune system. Therefore, Equations 6–8, 13–19 can be defined as the 10 standard conceptual equations of the tumor immuno-ecodynamics (summarized in **Figure 4**). Particularly, cancer patients have one more subsystem than normal people: the tumor ecosystem. Now, let us do a further thought experiment that requires the number of subsystems in a cancer patient to be equal to the number of subsystems in a healthy person. We can first try to incorporate the immune system into the tumor ecosystem, and it turns out that doing so makes the rules of operation of this combined subsystem very complex and ambiguous. For example, loading the positive and negative parts of the immune system into each of the 14 functional phenotypes (cancer hallmarks) of the tumor ecosystem yields a tumor ecosystem containing 28 ( 14×2=28) phenotypes. We then incorporated the tumor ecosystem into the immune system, and found that doing so made the rules of operation of the combined subsystems very simple and clear. Through this thought experiment, we can also argue that cancer patients do not have an extra subsystem, but that the negative part of their immune system is enhanced, and this negative part becomes more and more powerful as the disease progresses. This is based on the fact that, rather than subjectively assigning the tumor ecosystem to negative immunity, the tumor ecosystem has objectively hijacked the entire negative immunity. Tumor-related structures and phenotypes that appear to be unrelated to immunity can survive and be maintained only if they acquire the characteristics of negative immunity, otherwise they will be eliminated by positive immunity, because the tumor is not the tissue that the body needs. Therefore, the behavior of hijacking negative immunity is the inevitable requirement and underlying logic of the development of the tumor ecosystem. Because so far, only by doing so, can we use a clear immune balance law to deal with the interaction between the immune system and the tumor ecosystem, and can we establish a simple mathematical model that can be easily understood by medical researchers and clinicians. On the other hand, from a logical point of view, there is no reason to incorporate all the subsystems of healthy people into an emerging subsystem, the tumor ecosystem of cancer patients, and to do so would make the entire body system too complex to conduct precise studies. Similarly, in the same logical way, Schreiber et al. developed their theory of cancer immunoediting by (consciously or unconsciously) incorporating tumors (at that time, there was no concept or theory of tumor ecosystem) into the immune system, or by incorporating oncology into immunology (4, 5, 47). I have analyzed this in my previous published article (2). However, in current study, I mathematize this important theory for the first time. Therefore, it is logical to incorporate the theory of cancer immunoediting into the framework of tumor immuno-ecodynamics.

It is worth noting that the interaction between the two subsystems in this model is bidirectional, that is, the immune system suppresses the tumor ecosystem, and the tumor ecosystem also suppresses the immune system. Some other theories describing how the immune system interacts with the tumor ecosystem borrow from predator-prey relationships in natural ecosystems (53–55), where the relationship is one-way, with only the predator controlling the prey and the prey not possibly controlling the predator. For instance, the tumor ecosystem is not the food of the immune system. It is not the case that the immune system will become stronger as the tumor grows larger. Therefore, these theories are unable to truly reflect the essential connection between the immune system and the tumor ecosystem. However, there is a set of studies (56–60) that deserve attention. The authors established several mathematical models to describe the interaction between the immune system and tumors, and some of them incorporate therapeutic interventions, which are worth learning from. For instance, in future research, therapeutic interventions can be introduced to verify the validity and accuracy of the model for tumor immuno-ecosystem.

Throughout the study of the field, other tumor ecosystem theories based on a different way of thinking from the model established here describe the tumor ecosystem as a very complex system (7, 54, 55, 61–66). As a result, mathematical models of the tumor ecosystem built by logical methods that differ from this model are also very complex (67–72). These models invariably use sophisticated mathematical equations originally used to describe other systems and phenomena. For medical researchers and clinicians, these equations are very foreign and therefore difficult to be translated into guiding principles for clinical application. On the one hand, they have a hard time to understand these equations; on the other hand, it is difficult for them to confirm the applicability of these equations with experimental and clinical studies. In terms of methodology, only by incorporating the tumor ecosystem with ambiguous rules into the immune system with clear rules (equilibrium law) can we obtain a simplified mathematical model that reflects the essential laws of the system. Here, I advocate a new research direction, that is, to use the symbols of immunology and oncology to mathematize the existing theories of immunology and oncology through simplified rules, to establish professional and friendly mathematical models of immunology and oncology (or immune-oncology), and promote the clinical application of these models.

The model established in this article has some limitations. For instance, it regards tumor volume ( V) as a key parameter, but due to the heterogeneity of tumors (such as necrotic zones, stromal proportion, etc.), these heterogeneities can affect the assessment of tumor ecological momentum ( $M_{te}^{'}$). When sampling the same tumor focus before and after treatment in a study, it is necessary to take samples from the same area for comparison. How to avoid asymmetrical samples will be very important, and thus there may be practical difficulties in operation. Additionally, this highly simplified mathematical model may cause misunderstandings among readers. For example, Equations 6, 7 are composed of only four core immunodynamic factors (two core factor pairs), how can they represent the countless positive and negative factor pairs between the immune system and the tumor ecosystem? Although there is already proof from information theory equations, it seems that a strict proof using pure algebraic methods is still needed. In fact, through the analysis of first-order and second-order derivatives in calculus, monotonic mapping, and steady-state analysis of dynamical systems, it can be proven that Equations 1, 6, or Equations 2, 7, although not strictly equal in a pure algebraic sense, have completely consistent dynamic characteristics (change rate, curve shape, extreme points, and steady-state baseline), and belong to the equivalent description of biological dynamics. They can precisely characterize the entire process of immune response through the equations composed of the four core factors. However, the target readers of this article are medical researchers and clinicians, I decide not present these advanced mathematical methods, which require a considerable amount of space. If readers are interested in these mathematical proofs, they can contact me via email for further discussion.

## 4 Conclusion

In this study, the previously established immunodynamics and tumor ecodynamics are mathematically standardized, and incorporated into the framework of information theory, then integrated into the theory of tumor immuno-ecodynamics. This model unifies four important theories of immunology and oncology (or immuno-oncology), namely the theories of immune equilibrium, tumor ecosystem, cancer hallmarks and cancer immunoediting. Particularly, it can quantitatively describe the immune system, the tumor ecosystem and their interactions, and can be used to guide individualized cancer immunotherapy when combining with the previous established models (1, 2), representing an important step in transforming immunology and oncology from qualitative to quantitative sciences.

## Data Availability

The original contributions presented in the study are included in the article/supplementary material. Further inquiries can be directed to the corresponding author.

## References

[1] Chen X . From immune equilibrium to immunodynamics. Front Microbiol. (2022) 13:1018817. doi: 10.3389/fmicb.2022.1018817. PMID: 36504800 PMC9732466

[2] Chen X . From immune equilibrium to tumor ecodynamics. Front Oncol. (2024) 14:1335533. doi: 10.3389/fonc.2024.1335533. PMID: 38807760 PMC11131381

[3] Golan A Harte J . Information theory: A foundation for complexity science. Proc Natl Acad Sci USA. (2022) 119:e2119089119. doi: 10.1073/pnas.2119089119. PMID: 35895715 PMC9388134

[4] Dunn GP Old LJ Schreiber RD . The immunobiology of cancer immunosurveillance and immunoediting. Immunity. (2004) 21:137–48. doi: 10.1016/j.immuni.2004.07.017. PMID: 15308095

[5] Dunn GP Old LJ Schreiber RD . The three Es of cancer immunoediting. Annu Rev Immunol. (2004) 22:329–60. doi: 10.1146/annurev.immunol.22.012703.104803. PMID: 15032581

[6] Eberl G . Immunity by equilibrium. Nat Rev Immunol. (2016) 16:524–32. doi: 10.1038/nri.2016.75. PMID: 27396446

[7] Chen X Song E . The theory of tumor ecosystem. Cancer Commun Lond. (2022) 42:587–608. doi: 10.1002/cac2.12316. PMID: 35642770 PMC9257988

[8] Hanahan D . Hallmarks of cancer: New dimensions. Cancer Discov. (2022) 12:31–46. doi: 10.1158/2159-8290.cd-21-1059. PMID: 35022204

[9] Cover TM Thomas JA . Entropy, relative entropy, and mutual information. In: Elements of information theory. John Wiley & Sons, Inc. p. 13–55. doi: 10.1002/0471200611.ch2

[10] Lanier LL . Face off--the interplay between activating and inhibitory immune receptors. Curr Opin Immunol. (2001) 13:326–31. doi: 10.1126/science.290.5489.84. PMID: 11406364

[11] O'Shea JJ Holland SM Staudt LM . JAKs and STATs in immunity, immunodeficiency, and cancer. N Engl J Med. (2013) 368:161–70. doi: 10.1056/NEJMra1202117 PMC7604876 23301733

[12] Chen L Flies DB . Molecular mechanisms of T cell co-stimulation and co-inhibition. Nat Rev Immunol. (2013) 13:227–42. doi: 10.1038/nri3405. PMID: 23470321 PMC3786574

[13] O'Neill LAJ Kishton RJ Rathmell J . A guide to immunometabolism for immunologists. Nat Rev Immunol. (2016) 16:553–65. doi: 10.1038/nri.2016.70 PMC5001910 27396447

[14] Viganò S Perreau M Pantaleo G Harari A . Positive and negative regulation of cellular immune responses in physiologic conditions and diseases. J Immunol Res. (2012) 2012:485781. doi: 10.1155/2012/485781 PMC3324270 22548114

[15] Arneth B . Molecular mechanisms of immune regulation: A review. Cells. (2025) 14:283. doi: 10.3390/cells14040283. PMID: 39996755 PMC11853995

[16] Paludan SR Pradeu T Masters SL Mogensen TH . Constitutive immune mechanisms: mediators of host defence and immune regulation. Nat Rev Immunol. (2021) 21:137–50. doi: 10.1038/s41577-020-0391-5. PMID: 32782357 PMC7418297

[17] Feixas M Bardera A Rigan J Sbert M Xu Q . Information theory basics. In: Feixas M Bardera A Rigan J Sbert M Xu Q , editor.Information theory tools for image processing. Springer International Publishing, Cham (2014). p. 1–23.

[18] Han L Wu T Zhang Q Qi A Zhou X . Immune tolerance regulation is critical to immune homeostasis. J Immunol Res. (2025) 2025:5006201. doi: 10.1155/jimr/5006201. PMID: 39950084 PMC11824399

[19] Parijs LV Abbas AK . Homeostasis and self-tolerance in the immune system: Turning lymphocytes off. Science. (1998) 280:243–8. doi: 10.1126/science.280.5361.243. PMID: 9535647

[20] Chen L He Z Qin L Li Q Shi X Zhao S . Antitumor effect of malaria parasite infection in a murine Lewis lung cancer model through induction of innate and adaptive immunity. PloS One. (2011) 6:e24407. doi: 10.1371/journal.pone.0024407. PMID: 21931708 PMC3170332

[21] Yang Y Liu Q Lu J Adah D Yu S Zhao S . Exosomes from Plasmodium-infected hosts inhibit tumor angiogenesis in a murine Lewis lung cancer model. Oncogenesis. (2017) 6:e351. doi: 10.1038/oncsis.2017.52. PMID: 28650446 PMC5519199

[22] Liu Q Yang Y Tan X Tao Z Adah D Yu S . Plasmodium parasite as an effective hepatocellular carcinoma antigen glypican-3 delivery vector. Oncotarget. (2017) 8:24785–96. doi: 10.18632/oncotarget.15806. PMID: 28445973 PMC5421888

[23] Adah D Yang Y Liu Q Gadidasu K Tao Z Yu S . Plasmodium infection inhibits the expansion and activation of MDSCs and Tregs in the tumor microenvironment in a murine Lewis lung cancer model. Cell Commun Signal. (2019) 17:32. doi: 10.1186/s12964-019-0342-6. PMID: 30979375 PMC6461823

[24] Wang B Li Q Wang J Zhao S Nashun B Qin L . Plasmodium infection inhibits tumor angiogenesis through effects on tumor-associated macrophages in a murine implanted hepatoma model. Cell Commun Signal. (2020) 18:157. doi: 10.1186/s12964-020-00570-5. PMID: 32972437 PMC7513281

[25] Chen X Qin L Hu W Adah D . The mechanisms of action of Plasmodium infection against cancer. Cell Commun Signal. (2021) 19:74. doi: 10.1186/s12964-021-00748-5. PMID: 34243757 PMC8268363

[26] Pan J Ma M Qin L Kang Z Adah D Tao Z . Plasmodium infection inhibits triple negative 4T1 breast cancer potentially through induction of CD8(+) T cell-mediated antitumor responses in mice. BioMed Pharmacother. (2021) 138:111406. doi: 10.1016/j.biopha.2021.111406. PMID: 33676307

[27] Chen X Tao Z Liang Y Ma M Adah D Ding W . Plasmodium immunotherapy combined with gemcitabine has a synergistic inhibitory effect on tumor growth and metastasis in murine Lewis lung cancer models. Front Oncol. (2023) 13:1181176. doi: 10.3389/fonc.2023.1181176. PMID: 37916167 PMC10618005

[28] Tao Z Ding W Cheng Z Feng Y Kang Z Qiu R . Preclinical study of Plasmodium immunotherapy combined with radiotherapy for solid tumors. Cells. (2022) 11. doi: 10.3390/cells11223600. PMID: 36429033 PMC9688403

[29] Qin L Zhong M Adah D Qin L Chen X Ma C . A novel tumour suppressor lncRNA F630028O10Rik inhibits lung cancer angiogenesis by regulating miR-223-3p. J Cell Mol Med. (2020) 24:3549–59. doi: 10.1111/jcmm.15044. PMID: 32052546 PMC7131933

[30] Liang Y Chen X Tao Z Ma M Adah D Li X . Plasmodium infection prevents recurrence and metastasis of hepatocellular carcinoma possibly via inhibition of the epithelial-mesenchymal transition. Mol Med Rep. (2021) 23. doi: 10.3892/mmr.2021.12057. PMID: 33846776 PMC8025467

[31] Huang Q Yang D Tao Z Yu Z Ding W Tao N . Plasmodium infection fully activates the immune system in peripheral blood and tumor microenvironment in a murine Lewis lung cancer model. Front Mol Biosci. (2026) 12:2025. doi: 10.3389/fmolb.2025.1724792. PMID: 41685208 PMC12892102

[32] Shi X Qin L Liu G Zhao S Peng N Chen X . Dynamic balance of pSTAT1 and pSTAT3 in C57BL/6 mice infected with lethal or nonlethal Plasmodium yoelii. Cell Mol Immunol. (2008) 5:341–8. doi: 10.1038/cmi.2008.42. PMID: 18954557 PMC4073698

[33] Xie Y Wang J Wang Y Wen Y Pu Y Wang B . Parasite-enhanced immunotherapy: Transforming the “cold” tumors to “hot” battlefields. Cell Commun Signaling. (2024) 22:448. doi: 10.1186/s12964-024-01822-4. PMID: 39327550 PMC11426008

[34] Eissa MM Salem AE El Skhawy N . Parasites revive hope for cancer therapy. Eur J Med Res. (2024) 29:489. doi: 10.1186/s40001-024-02057-2. PMID: 39367471 PMC11453045

[35] Dobosz P Dzieciątkowski T . The intriguing history of cancer immunotherapy. Front Immunol. (2019) 10:2965. doi: 10.3389/fimmu.2019.02965. PMID: 31921205 PMC6928196

[36] Munir M Cheema AY Ogedegbe OJ Aslam MF Kim S . William Coley: The pioneer and the father of immunotherapy. Cureus. (2024) 16:e69113. doi: 10.7759/cureus.69113. PMID: 39391466 PMC11466495

[37] Pettenati C Ingersoll MA . Mechanisms of BCG immunotherapy and its outlook for bladder cancer. Nat Rev Urol. (2018) 15:615–25. doi: 10.1038/s41585-018-0055-4. PMID: 29991725

[38] Kaufman HL Kohlhapp FJ Zloza A . Oncolytic viruses: A new class of immunotherapy drugs. Nat Rev Drug Discov. (2015) 14:642–62. doi: 10.1038/nrd4663. PMID: 26323545 PMC7097180

[39] Lin D Shen Y Liang T . Oncolytic virotherapy: Basic principles, recent advances and future directions. Signal Transduc Tgt Ther. (2023) 8:156. doi: 10.1038/s41392-023-01407-6. PMID: 37041165 PMC10090134

[40] Larocca C Schlom J . Viral vector-based therapeutic cancer vaccines. Cancer J. (2011) 17:359–71. doi: 10.1097/ppo.0b013e3182325e63. PMID: 21952287 PMC3207353

[41] Marelli G Howells A Lemoine NR Wang Y . Oncolytic viral therapy and the immune system: A double-edged sword against cancer. Front Immunol. (2018) 9:866. doi: 10.3389/fimmu.2018.00866. PMID: 29755464 PMC5932159

[42] Meng L Wu H Wu J Ding PA He J Sang M . Mechanisms of immune checkpoint inhibitors: Insights into the regulation of circular RNAS involved in cancer hallmarks. Cell Death Dis. (2024) 15:3. doi: 10.1038/s41419-023-06389-5. PMID: 38177102 PMC10766988

[43] Hargadon KM Johnson CE Williams CJ . Immune checkpoint blockade therapy for cancer: An overview of FDA-approved immune checkpoint inhibitors. Int Immunopharmacol. (2018) 62:29–39. doi: 10.1016/j.intimp.2018.06.001. PMID: 29990692

[44] Krysko DV Garg AD Kaczmarek A Krysko O Agostinis P Vandenabeele P . Immunogenic cell death and DAMPs in cancer therapy. Nat Rev Cancer. (2012) 12:860–75. doi: 10.1038/nrc3380. PMID: 23151605

[45] Yu H Pardoll D Jove R . STATs in cancer inflammation and immunity: A leading role for STAT3. Nat Rev Cancer. (2009) 9:798–809. doi: 10.1038/nrc2734. PMID: 19851315 PMC4856025

[46] Hu R Han Q Zhang J . STAT3: A key signaling molecule for converting cold to hot tumors. Cancer Lett. (2020) 489:29–40. doi: 10.1016/j.canlet.2020.05.035. PMID: 32522692

[47] Wu T Wang G Wang X Wang S Zhao X Wu C . Quantification of neoantigen-mediated immunoediting in cancer evolution. Cancer Res. (2022) 82:2226–38. doi: 10.1158/0008-5472.can-21-3717. PMID: 35486454

[48] Qin L Chen C Chen L Xue R Ou-Yang M Zhou C . Worldwide malaria incidence and cancer mortality are inversely associated. Infect Agents Cancer. (2017) 12:14. doi: 10.1186/s13027-017-0117-x. PMID: 28228842 PMC5307699

[49] Hu W Tao Z Guo W Ding W Huang S Li J . Protein catenation potentiates antitumor activity of malaria VAR2CSA navigation CAR-T cells in a mouse model of hematological Malignancies. J Immunother Cancer. (2025) 13:e012618. doi: 10.1136/jitc-2025-012618. PMID: 41260900 PMC12636975

[50] Swanton C Bernard E Abbosh C André F Auwerx J Balmain A . Embracing cancer complexity: Hallmarks of systemic disease. Cell. (2024) 187:1589–616. doi: 10.1016/j.cell.2024.02.009. PMID: 38552609 PMC12077170

[51] Sibai M Cervilla S Grases D Musulen E Lazcano R Mo C-K . The spatial landscape of cancer hallmarks reveals patterns of tumor ecological dynamics and drug sensitivity. Cell Rep. (2025) 44:115229. doi: 10.1158/1538-7445.am2025-lb265. PMID: 39864059

[52] Bhattacharya R Avdieiev SS Bukkuri A Whelan CJ Gatenby RA Tsai KY . The hallmarks of cancer as eco-evolutionary processes. Cancer Discov. (2025) 15:685–701. doi: 10.1158/2159-8290.cd-24-0861. PMID: 40170539

[53] Kareva I Luddy KA O’Farrelly C Gatenby RA Brown JS . Predator-prey in tumor-immune interactions: a wrong model or just an incomplete one? Front Immunol. (2021) 12:668221. doi: 10.3389/fimmu.2021.668221. PMID: 34531851 PMC8438324

[54] Hamilton PT Anholt BR Nelson BH . Tumour immunotherapy: lessons from predator–prey theory. Nat Rev Immunol. (2022) 22:765–75. doi: 10.1038/s41577-022-00719-y. PMID: 35513493

[55] Perret C Gidoin C Ujvari B Thomas F Roche B . Predation shapes the impact of cancer on population dynamics and the evolution of cancer resistance. Evol Appl. (2020) 13:1733–44. doi: 10.1111/eva.12951. PMID: 32821280 PMC7428821

[56] Necati Ö Esmehan U . Investigating of an immune system-cancer mathematical model with Mittag-Leffler kernel. AIMS Math. (2020) 5:1519–31. doi: 10.3934/math.2020104

[57] Uçar E . Examining of a tumor system with Caputo derivative. Balıkesir Üniversitesi Fen Bilimleri Enstitüsü Dergisi. (2023) 25:37–48. doi: 10.25092/baunfbed.1113646

[58] Uçar E Özdemir N . New fractional cancer mathematical model via IL-10 cytokine and anti-PD-L1 inhibitor. Fractal Fractional. (2023) 7:151. doi: 10.3390/fractalfract7020151. PMID: 30654563

[59] Uçar E Özdemir N Altun E . Qualitative analysis and numerical simulations of new model describing cancer. J Comput Appl Math. (2023) 422:114899. doi: 10.1016/j.cam.2022.114899

[60] Uçar S Koca I Özdemir N İnci T . A stochastic approach to tumor modeling incorporating macrophages. Bull Biomath. (2024). doi: 10.59292/bulletinbiomath.2024007

[61] Kareva I . Cancer ecology: niche construction, keystone species, ecological succession, and ergodic theory. Biol Theory. (2015) 10:283–8. doi: 10.1007/s13752-015-0226-y. PMID: 30311153

[62] Pienta KJ McGregor N Axelrod R Axelrod DE . Ecological therapy for cancer: defining tumors using an ecosystem paradigm suggests new opportunities for novel cancer treatments. Transl Oncol. (2008) 1:158–64. doi: 10.1593/tlo.08178. PMID: 19043526 PMC2582164

[63] Korolev KS Xavier JB Gore J . Turning ecology and evolution against cancer. Nat Rev Cancer. (2014) 14:371–80. doi: 10.1038/nrc3712. PMID: 24739582 PMC13213539

[64] Merlo LMF Pepper JW Reid BJ Maley CC . Cancer as an evolutionary and ecological process. Nat Rev Cancer. (2006) 6:924–35. doi: 10.1038/nrc2013. PMID: 17109012

[65] Zahir N Sun R Gallahan D Gatenby RA Curtis C . Characterizing the ecological and evolutionary dynamics of cancer. Nat Genet. (2020) 52:759–67. doi: 10.1038/s41588-020-0668-4. PMID: 32719518

[66] Reynolds BA Oli MW Oli MK . Eco-oncology: applying ecological principles to understand and manage cancer. Ecol Evol. (2020) 10:8538–53. doi: 10.1002/ece3.6590. PMID: 32884638 PMC7452771

[67] Altrock PM Liu LL Michor F . The mathematics of cancer: integrating quantitative models. Nat Rev Cancer. (2015) 15:730–45. doi: 10.1038/nrc4029. PMID: 26597528

[68] Serre R Benzekry S Padovani L Meille C André N Ciccolini J . Mathematical modeling of cancer immunotherapy and its synergy with radiotherapy. Cancer Res. (2016) 76:4931–40. doi: 10.1158/0008-5472.can-15-3567. PMID: 27302167

[69] Yin A Moes D van Hasselt JGC Swen JJ Guchelaar HJ . A review of mathematical models for tumor dynamics and treatment resistance evolution of solid tumors. CPT Pharmacomet Syst Pharmacol. (2019) 8:720–37. doi: 10.1002/psp4.12450. PMID: 31250989 PMC6813171

[70] Butner JD Wang Z Elganainy D Al Feghali KA Plodinec M Calin GA . A mathematical model for the quantification of a patient’s sensitivity to checkpoint inhibitors and long-term tumour burden. Nat BioMed Eng. (2021) 5:297–308. doi: 10.1038/s41551-020-00662-0. PMID: 33398132 PMC8669771

[71] Butner JD Dogra P Chung C Pasqualini R Arap W Lowengrub J . Mathematical modeling of cancer immunotherapy for personalized clinical translation. Nat Comput Sci. (2022) 2:785–96. doi: 10.1038/s43588-022-00377-z. PMID: 38126024 PMC10732566

[72] Aguadé-Gorgorió G Anderson ARA Solé R . Modeling tumors as complex ecosystems. iScience. (2024), 110699. doi: 10.1016/j.isci.2024.110699 39280631 PMC11402243

